# A High Quality Asian Genome Assembly Identifies Features of Common Missing Regions

**DOI:** 10.3390/genes11111350

**Published:** 2020-11-13

**Authors:** Jina Kim, Joohon Sung, Kyudong Han, Wooseok Lee, Seyoung Mun, Jooyeon Lee, Kunhyung Bahk, Inchul Yang, Young-Kyung Bae, Changhoon Kim, Jong-Il Kim, Jeong-Sun Seo

**Affiliations:** 1 Interdisciplinary Program of Bioinformatics, College of Natural Science, Seoul National University, Seoul 08826, Korea; neutrogena@snu.ac.kr (J.K.); kage0517@snu.ac.kr (K.B.); 2 Genome & Health Big Data Laboratory, Department of Health Science, Seoul National University, Seoul 08826, Korea; 3 Institute of Health & Environment, Seoul National University, Seoul 08826, Korea; esterita@snu.ac.kr; 4 DKU-Theragen institute for NGS analysis (DTiNa), Cheonan 31116, Korea; wooseoklee87@gmail.com (W.L.); munseyoung@gmail.com (S.M.); 5 Center for Bio-Medical Engineering Core Facility, Dankook University, Cheonan 31116, Korea; 6 Department of Microbiology, Dankook University, Cheonan 31116, Korea; 7 Department of Nanobiomedical Science, Dankook University, Cheonan 31116, Korea; 8 Center for Bio-Analysis, Korea Research Institute of Standards and Science, Daejeon 34113, Korea; icy@kriss.re.kr (I.Y.); ybae@kriss.re.kr (Y.-K.B.); 9 Bioinformatics Institute, Macrogen Inc., Seoul 08511, Korea; kimchan@macrogen.com (C.K.); jeongsunseo@gmail.com (J.-S.S.); 10 Genomic Medicine Institute, Medical Research Center, Seoul National University, Seoul 03080, Korea; jongil@snu.ac.kr; 11 Department of Biochemistry and Molecular Biology, Seoul National University College of Medicine, Seoul 03080, Korea; 12 Precision Medicine Center, Seoul National University Bundang Hospital, Seongnam 13605, Korea; 13 Gong-Wu Genomic Medicine Institute, Seoul National University Bundang Hospital, Seongnam 13605, Korea

**Keywords:** missing information, human reference genome, precise ethnic genome, occurrence mechanism

## Abstract

The current human reference genome (GRCh38), with its superior quality, has contributed significantly to genome analysis. However, GRCh38 may still underrepresent the ethnic genome, specifically for Asians, though exactly what we are missing is still elusive. Here, we juxtaposed GRCh38 with a high-contiguity genome assembly of one Korean (AK1) to show that a part of AK1 genome is missing in GRCh38 and that the missing regions harbored ~1390 putative coding elements. Furthermore, we found that multiple populations shared some certain parts in the missing genome when we analyzed the “unmapped” (to GRCh38) reads of fourteen individuals (five East-Asians, four Europeans, and five Africans), amounting to ~5.3 Mb (~0.2% of AK1) of the total genomic regions. The recovered AK1 regions from the “unmapped reads”, which were the estimated missing regions that did not exist in GRCh38, harbored candidate coding elements. We verified that most of the common (shared by ≥7 individuals) missing regions exist in human and chimpanzee DNA. Moreover, we further identified the occurrence mechanism and ethnic heterogeneity as well as the presence of the common missing regions. This study illuminates a potential advantage of using a pangenome reference and brings up the need for further investigations on the various features of regions globally missed in GRCh38.

## 1. Introduction

DNA sequencing of the human genome is the basis of precision medicine. An overwhelming majority of sequencing performs resequencing of massive short reads, using the GRCh37/38 (a.k.a., hg19/38) human genome assembly as a reference. GRCh38 is the successor of the Human Genome Project. The GRCh38 has been further enriched (~30%) by the addition of genomes from >50 individuals, including contributions from those of African ancestry [1]. The general belief has been that a single global reference genome was sufficient because resequencing requires a reference to determine the genetic variants of individuals, rather than a database encompassing a list of variants. However, researchers find it substantial that recent findings point out the diversity of structural variation among ethnic groups [2,3] and that the human migration has evolved into complex detours. This pattern of human migration resulted in local admixture [4] and has led to questions about whether some portions of the DNA sequences are missed by the current re-sequencing methods [5,6]. In attempts to find the missing regions, some previous studies have used the “unmapped” reads, which are sequence reads of the RNA sequencing data [7] and DNA sequencing data [8,9,10] that fail to align to the reference, to identify regions with suggestive evidence of protein coding [8] or disease association [9] on the previous reference version and GRCh38. Researchers used raw fragmented genome data, and performed de novo assembly of the unmapped reads of different individuals for comparison with the reference [8]. However, when the short reads were assembled into the contigs, the contigs created from the short reads could have had missing or limited information due to the lack of continuity compared to the contigs from long reads, which raised the difficulty of placing the contigs in the reference genome.

In addition to using the de novo assembly of short unmapped reads, other studies found missing regions with long read sequences relative to GRCh38 and thoroughly examined the possibility of using the sequences as a reference patch to discover structural variants [11] and alternate alleles [12]. Although the common missing regions explored in several studies represented structural variations and alternate alleles that are not on the GRCh38 reference genome and discussed the potential of “pan reference”, the gap in literature that studies the occurrence mechanism of common missing regions should be highlighted.

In this study, we first performed a comparison of the two human genome assemblies, GRCh38 and AK1 (one Korean genome assembly) [13], with high contiguity, and outlined the differences between the two genomes. Second, we re-aligned the “unmapped” reads of general samples to new assembly and further specified the estimated missing parts by tracing re-aligned “unmapped” reads. Finally, we also searched for the putative functions of the missing reference sequence and investigated the mechanisms of these events by experimentally verifying the presence and characteristics of the missing regions.

## 2. Materials and Methods

### 2.1. Comparison between the Reference Genome (GRCh38) and the AK1

Our study was approved by the Institutional Review Board of Seoul National University (SNU 19-11-064). We used the LASTZ program [14] to generate a chain file between the AK1 (GCA_001750385.2) and GRCh38 (GCA_000001405.27) downloaded from NCBI website (Available online: https://www.ncbi.nlm.nih.gov/assembly/ (accessed on 21 July 2018)), with written parameters (--gapped --gap = 600, 150, --hspthresh = 4500, --seed = 12 of 19 --notransition --ydrop = 15,000) [10].

**Figure 1:**
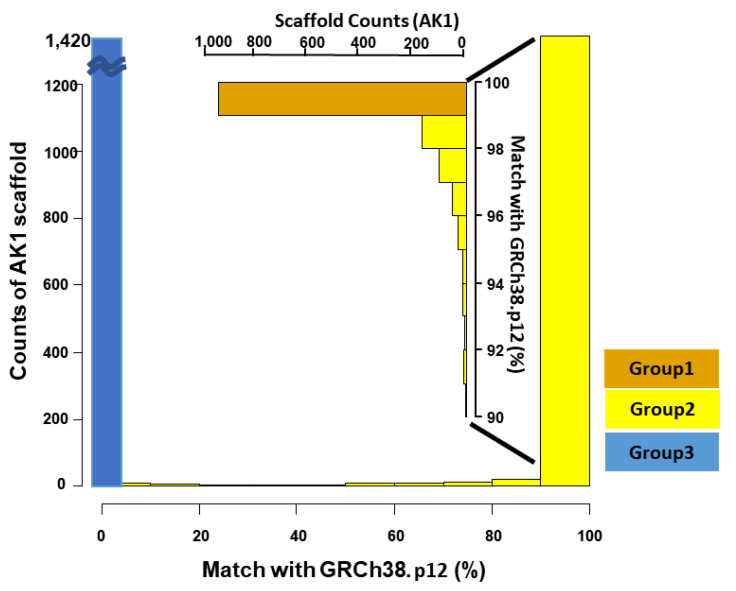
A systematic comparison between AK1 scaffolds (*n* = 2382) and GRCh38.p12. The degree of match divided AK1 scaffolds into three distinct patterns of synteny by LASTZ [14]. The x axis (and vertical pop-up axis for Group 1) represents the percent of matches between AK1 scaffold and GRCh38.p12 chromosomes, and the y axis represents the count of scaffolds. GRCh38.p12, Genome Reference Consortium Human Build 38 patch release 12.

We used UCSC Kent utilities (Available online: https://github.com/ENCODE-DCC/kentUtils (accessed on 14 November 2018)) for the chaining and netting process. By generating bidirectional “chain files” indicating both homology and gaps at base-pair resolution, we categorized a total of 2832 scaffolds of AK1 into three groups according to the alignment patterns (Figure 1).

Group 1: The first scaffold group (*n* = 945, ~2.70 Gbp in total) consisted of ≥99% of the chromosomes of GRCh38.

Group 2: The second group (*n* = 467, ~165 Mb in total) presented partial (0% < X < 99%) matches.

Group 3: The third scaffold group (*n* = 1420, ~41 Mb) lacked synteny with GRCh38.

Based on the synteny of and gaps in the chain file, we calculated the alignments between the AK1 scaffolds and GRCh38 chromosomes. To strengthen the reliability of our LASTZ results, we randomly selected a scaffold in each group, and appointed the parameters as set 1. We performed a comparison analysis using different parameters and randomly selected scaffolds (Table S1). As a result, the chain files of the scaffolds using other parameters were not significantly different.

### 2.2. Study Samples and Materials for the Profiling of Sequencing Reads to the Reference Genome (GRCh38)

To extract unmapped reads from bam files aligned to GRCh38, the bam files of all samples, which were already aligned to the GRCh38 full analysis set with HLA sequences, were downloaded from the 1000 Genomes browser [2]. All the WGS data were generated on HiSeq platforms (Illumina, Sandiego) with PCR-free procedures. We only selected 14 samples from 3 ethnic groups, which were deeply sequenced (depth > X50), mapped to GRCh38 with BWA-MEM (version bwakit-0.7.12.) [15], and subjected to the specified written QC processes (Available online: ftp://ftp.1000genomes.ebi.ac.uk/vol1/ftp/data_collections/1000_genomes_project (accessed on 12 December 2018)) including sorting, marking duplicates, and Indel realignment by SAMtools (version 1.2), BioBamBam (version 0.0.191) [16], GATK-3.3-0 [17] and CRAMtools.3.0. 

### 2.3. Investigation of the Characteristics of Mapped/Unmapped Reads from BAM Files and Realignment of the Extracted “Unmapped Reads” against the AK1 Genome Assembly

**Figure 2:**
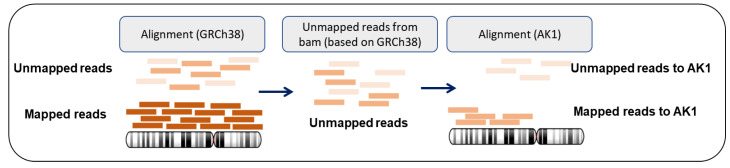
The process of realigning unmapped reads of GRCh38 to AK1.

For quality checks and repetitive annotation of the mapped/unmapped reads, we used FastQC [18] and RepeatMasker [19]. After samtools (samtools view -b -f 4 Inputfile) was used to extract the unmapped reads from the downloaded BAM files of the 14 multiethnic samples, we realigned the unmapped reads to the AK1 genome assembly using BWA-MEM (version 0.7.17-r1188) (Figure 2). After realignment, the sorting and the removal of duplicates were performed by SAMtools (version 1.3) and Picard Tools (version 2.0.1).

We only used reads of primary alignments to exclude reads aligning reasonably well to more than one place. We calculated the depth/breadth [20] and excluded regions with low depths(<3×) from realigned bam files for each individual using BEDTools (version 2.25.0) [21] and Samtools (version 1.3). We used output data from BEDTools to show coverage and count depth by genomic positions with R (version 3.4.3). We also used GATK-pathSeq [22] to identify those reads including the putative microbial sequences.

### 2.4. Functional Search for the Common Missing Regions

We searched for functional clues via BLASTx [23] for the sequences (>200 bp) that were shown to be unique to AK1 when compared with GRCh38. Additionally, we performed both BLASTn (with an e-value < 10^−10^, identity ≥70%, and coverage ≥70%) and BLASTx (with an e-value < 10^−10^, identity ≥ 70%, and alignment length ≥50 bp) searches against the nr database with default options to find whether the estimated missing regions on AK1, which were re-aligned with more than ten “unmapped reads” from bam files of two or more individuals, exist across populations, and to speculate the functional roles of the missing regions.

For further investigation on the candidate regions located on Group 1 scaffolds that are missing globally, which is defined as common missing regions in seven or more individuals, we searched the locations of the missing regions in the GRCh38 genome using a chain file (“lifting” AK1 over GRCh38). To visualize these regions, we merged 14 BAM files into one and used the UCSC genome browser [24] and Integrative Genomics Viewer (IGV) [25] to visualize the merged BAM file. The suggested functional roles of the globally missing regions were also identified via BLASTx searches with an e-value < 10^−10^, identity ≥ 70%, and alignment length ≥ 50 bp.

### 2.5. Verification of the Missing Regions by PCR

After the functional comparison, we selected ±2 kb of the flanking sequences on the AK1 genome from the candidate regions that are missing globally in seven or more individuals to verify the existence of the regions and also to put them into the BLAST-Like Alignment Tool (BLAT) (Available online: http://genome.ucsc.edu/cgi-bin/hgBlat (accessed on 4 May 2019)) for human (GRCh38; December 2013) and chimpanzee (panTro6; February 2018) genomes [26] to investigate the characteristics of the globally missing common regions on Group 1 scaffolds. For the experimental confirmation of the non-overlapping AK1 sequences, we performed PCR amplification using four European DNA samples and a chimpanzee DNA sample, which was distributed by the Coriell Institute (Coriell Cell Repository, Camden, NJ, USA) and provided by Dr. Takenaka (Primate Research Institute, Kyoto University, Japan). The following cell lines/DNA samples were obtained from the NIGMS Human Genetic Cell Repository at the Coriell Institute for Medical Research: NA17001, NA17002, NA17003, and NA17004. The oligonucleotide primers used for the PCR amplification of each locus were designed by using the software Primer3 [27]. The PCR amplification of each locus was conducted with 25 uL of the reaction using 100 ng of DNA, 10 μL of 2X Lamp Pfu DNA polymerase (BioFact, Daejeon, Korea), and 10 pmol/μL of each oligonucleotide primer. The PCR conditions were as follows: 95 °C for 5 min, followed by 35 cycles of 30 sec of denaturation at 95 °C, 40 s at the annealing temperature, and 1 to 7 min of extension at 72 °C (depending on the expected size of the PCR product), followed by a final 5 min extension at 72 °C. Specific primer designs for 11 loci out of 31 putative insertions could not be done due to the absence of their sequence counterparts in the UCSC reference genome and the abundance of simple repeats and tranposable elements.

## 3. Results

### 3.1. Systematic Comparison between GRCh38 p.12 and AK1

We first compared the whole AK1 sequence against GRCh38 (“liftover”) and its alternative sequences to search for synteny. A total of 53.4 Mb (~1.8%) of the AK1 genome lacks homology with GRCh38, as we calculated the difference between “Total scaffold size” and “Size matched with GRCh38.p12” in Table 1. Dividing GRCh38 genome sequences by sequence types (chromosome; fix, error corrections or assembly improvements applied to the GRCh38 genome; random, the unlocalized contigs; unknown chromosome), we also investigated the matching sizes between AK1 scaffolds and GRCh38 genome by sequence type. The Group 1 and 2 scaffolds of AK1 matched with multiple chromosomes of GRCh38, among which the contributions of ectopic chromosomes amounted to ~22.2 Mb (~0.76%). The third group of scaffolds, which are unique to AK1, presented different genome sequences and repeat components according to the analysis using the RepeatMasker [19]. Satellites, which are multiple copies of repeated patterns that can vary in length from a single base to several thousand bases, were predominant in the repetitive components of Group 3 scaffolds (Table S2). In addition, the majority of the small size scaffolds on the AK1 genome were grouped to the third group and the N50 of the third group was 34.6 kb, although the N50 of AK1 genome data from NCBI was 44.85 Mb. To identify genome sequences that were unique to AK1 compared to GRCh38, we selected 3333 regions larger than 200 bp and searched for putative protein-coding functions via a translated BLAST [23] search within mammals. A total of 1390 regions (e-value < 10^−10^, identity ≥ 70%, and alignment length ≥ 50 bp) were predicted to harbor putative protein-coding elements (Table S3).

### 3.2. Profile of the “Unmapped Reads”

We selected high-depth (>50×) WGS data of 14 individuals from the 1kG database comprising Caucasians (four individuals), Asians (five individuals), and Africans (five individuals). The data represented populations from different areas and were initially aligned against GRCh38 according to the specifically written quality control information (all from the Illumina HiSeq platform). On average, ~4.7% of the WGS data (~2.6 M out of 54.6M total reads per individual) failed to align with GRCh38 and its alternative sequences. The data of Africans had the lowest alignment, and that of Caucasians had the highest mapping rate to GRCh38 (Table 2). The quality score of the reads re-aligned to AK1 was 7.2, which was higher than that of the overall unmapped reads, and the reads to AK1 have compatible quality in terms of base quality and mapping quality, with slightly lower coverage compared with the reads that were initially mapped to GRCh38 (Figure S1, Table S4).

Meanwhile, the “unpaired reads” explained the most substantial part of the unmapped reads (~59%) (Figure S2) due to the differences in sequencing quality between read 1 and read 2. In addition to the generally lower sequencing quality, the proportion of repetitive sequences among unmapped reads showed approximately ten times more low-complexity and >2 times more simple repeats and satellites, presenting much lower proportions of SINEs, LINEs, and LTRs compared to the reference genome (Table S5). The use of massive, fragmented reads will inevitably generate equivocal data for alignment, particularly in satellite or low-complexity regions. Given the quality and components involved, technical characteristics intrinsic to the analytic platform rather than the incompleteness of the reference genome are likely to have given rise to the majority of the unmapped reads.

### 3.3. Genomic Regions Recovered by “Realignment” to AK1

On average, 71 K of the ~2.6 M reads per individual (mapping quality >10) were newly mapped to AK1, with a very small proportion of reads of microbial origins. The recovery rates from realignment to AK1 were relatively low (0.92% or 0.49% overall for high-fidelity mapping quality) and did not show substantial differences between populations (Table 2). The regions with recovered reads accounted for ~0.2% (5.3 Mb) of the AK1 genome. We classified the recovered reads by mapping the scaffolds into three groups as shown in Figure 1. The Group 1 scaffolds harbored the largest number (*n* = 58,340) of realigned reads; proportionally, however, the Group 3 scaffolds were populated more broadly with unmapped reads (Table S6). Most of the realignments occurred within putative insertions and absent regions on the GRCh38 genome as the depth of the regions have similarity with that of GRCh38 (Table S4). Our findings suggest that the addition of an ethnic reference allows some missing genome regions to be salvaged, although only a small portion of the “unmapped reads” was responsible for these results.

### 3.4. Characterization and Heterogeneity of Common Missing Parts

By realigning “unmapped reads” to AK1, we selected 110 regions (shared by ≥ 2 individuals with read depth ≥ X10 for each) and 38 regions (shared by ≥ 7 individuals with read depth ≥ X10 for each) as the estimated missing regions, which were not on GRCh38. We took a look into the characteristics of the AK1 regions by finding repetitive sequences. The proportion of SINE and LINE was a little higher in these regions, and the value of simple repeats and low complexity on 38 regions is about 11 percent (Table 3). In addition, we scrutinized the recovered regions with short unmapped reads in the public database. Sixty-four of the 110 regions were previously reported or exhibited homology in the BLASTn searches of the mammalian genome database [9,28,29] (Table S7). Notably, 25 regions showed putative mammalian protein-coding functions in the translated BLAST search on NCBI’s nr database (e-value < 10^−10^, identity ≥70%, and alignment length ≥50 bp). The list of the regions showing putative protein-coding functions is presented in Table S8. When we observed 38 regions selected as both globally sharing (≥7 individuals with read depth ≥ X10 for each) and commonly missing, one of the 38 regions was suggested to be highly homologous to *zinc finger protein 454 isoform 2* (Table S9).

The Group 1 scaffolds harbored 31 out of 38 common missing regions; the 31 regions could be visualized in comparison with GRCh38 and the flanking sequences were annotated. Typically, these regions were flanked by several repeat elements, such as *Alu* or *LINE* elements (Figure S3).

**Figure 3:**
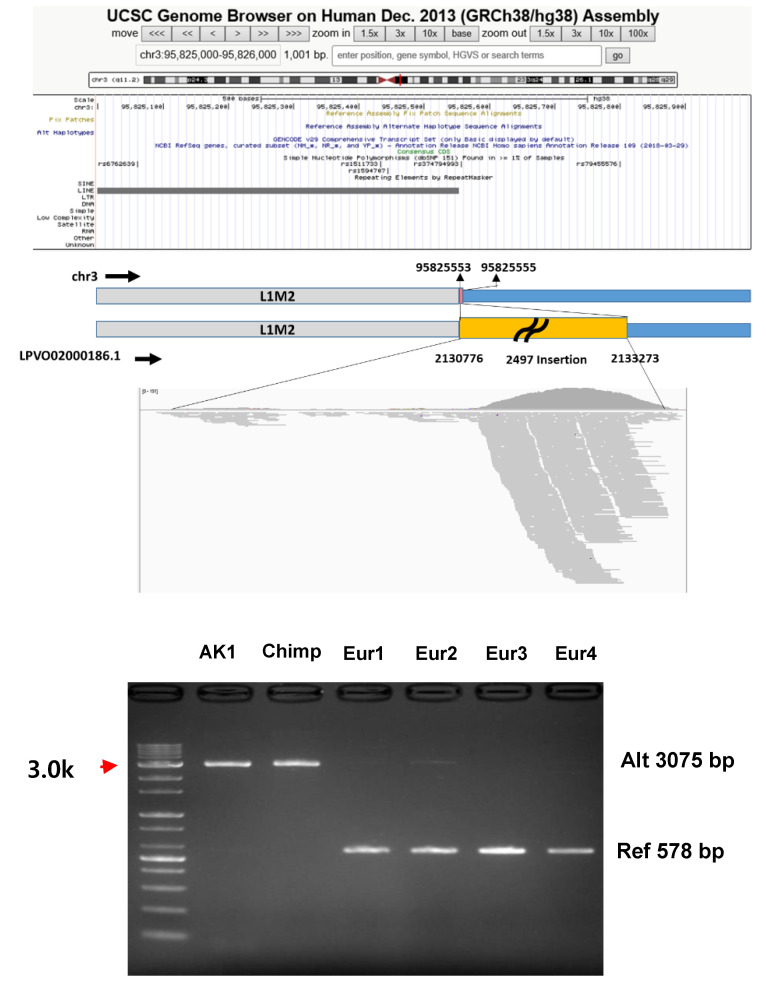
The example of globally missing regions on GRCh38 investigated with UCSC Genome browser and the experimental verification of the existence of the regions. The region (Group 1) with a high depth with 7 or more samples was discovered in the inserted sequences (yellow block). The G1-26 region (Insertion into chr3:95,825,553–95,825,555) was near L1M2. The yellow block is the estimated insertion against GRCh38 on the chain file. The grey blocks are repetitive sequences. The pink block is the sequence only on the GRCh38 genome. Chimp, chimpanzee; Eur, European.

After the functional comparison, we selected ±2 kb of the flanking sequences of the 38 regions to verify the existence of the regions. We experimentally verified the presence of the above 31 regions on Group 1 scaffolds, which were located within known locations of the reference genome. We conducted PCR amplification using the DNA of AK1, four Europeans and a chimpanzee. For AK1, 20 out of 31 putative insertions were verified, and 9 regions were also verified for the chimpanzee. Further examination of the breakpoints using BioEdit [30] suggested that nonhomologous end-joining with microhomology (NHEJ, *n* = 26) was the dominant occurrence mechanism followed by nonallelic homologous recombination (NAHR, *n* = 3). Interestingly, 26 of the 31 putative insertions presented exact matches with chimpanzee, and similar findings were obtained for the gorilla reference genome. For some regions, the Europeans subjects were either homozygous or heterozygous for insertions/deletions (Table 4). For example, the region (LPVO02000186.1: 2,132,760–2,132,810) on a scaffold of Group 1 was verified as insertion on GRCh38 (chr3: 95,822,539–95,830,080). In spite of the exsitence of missing region among AK1 (Korean), European and chimp, the inserted region was identified as a polymorphic region in European samples (Figure 3).

## 4. Discussion

A comparison between the reference genome and the precise ethnic genome suggested that the genomic differences between individuals exceed the previous consensus of “99.9% sharing” (which was primarily derived from human genome variation projects) and are far below in similarity with a 10% difference, derived from the assembly of unmapped reads of individuals of African ancestry [8]. This result may be explained by the fact that our results are derived from a two-genome comparison. Thus, the magnitude of the difference of ~1.8% might be either conservative or inflated: it may be conservative considering that GRCh38 is a composite genome from the contribution of >50 individuals and that structural variation was not considered, while it may be inflated considering that some of the satellite sequences showing a high proportion of repetitive sequences on Group 3 scaffolds, which are small in size, might not have been fully identified in the two assemblies.

However, it is unlikely that both possibilities have substantially affected the estimation of a ~1.8% difference, considering the quality of the two genome assemblies. In contrast to the estimated difference, only a portion of the “missing information” was recovered from the unmapped reads (<0.2% of AK1 sequences). It is likely that the differences are attributable to the high proportion of repetitive sequences in unique AK1 regions and the intrinsic limitations of the sequencing platform (e.g., extremely large numbers of low complexity characterized by the unmapped reads).

In addition, the analytic platforms for the de novo assembly between GRCh38 and AK1 differed mainly due to the time gap and rapid technological transition between the two assemblies. It is not likely that our findings reflect the methodological differences between the two genome assemblies, because we mainly focused on the regions that were confirmed through multiple approaches, which included laboratory testing.

Meanwhile, our study revealed that some parts of the missing regions might be common globally and harbor functional regions. According to our research concerning the characterization and heterogeneity of the common missing parts, the majority of the “globally missing” candidate regions found with unmapped reads of various populations might be deletions in the reference, rather than insertions in other populations. This result is consistent with a previous observation [11]. The functional search for the globally missing regions conducted in this study was preliminary and was limited to the coding sequences, so the suggested functional candidates require further validation. In addition to the existence and function of the missing parts, each of the missing parts have heterogeneity of the genomic structure by ethnicity and occurred by different mechanisms. This implies that there are differences in the occurrence mechanisms and structures in those common missing regions, although they were found in several population genomes. Thus, we see the necessity to further investigate the ethnically specific heterogenous structures and different occurrence mechanisms in the “commonly” missing regions.

In this study, we only used short reads for mapping to GRCh38 and also to AK1. Because some of the unmapped reads to both assembly genomes might be influenced by using short reads, the use of long read sequencing data could also help reduce the number of unmapped reads that stem from alignment ambiguities [31,32]. In addition, we did not compare recent pangenome and AK1, so that some of the “newly identified regions” in AK1 might overlap the pangenome of the Human Pangenome Reference Consortium (HPRC).

In conclusion, our study corroborates the usefulness of precise ethnic genomes for acquiring missing genomic information. Precise ethnic genomes in particular will become easier to obtain in the future and bear greater importance for understanding complete genome functions in addition to having a precise evolutionary history of humans. Precise ethnic genomes will also play an important role in finding other missing information and redress the research gap between populations.

## Figures and Tables

**Table 1:** Statistics of the three groups of AK1 scaffolds according to a systematic comparison between AK1 scaffolds (*n* = 2382) and GRCh38.p12. Fix, the patches represent changes (error corrections or assembly improvements) to GRCh38 genome; Random, the unlocalized contigs of GRCh38; GRCh38.p12, Genome Reference Consortium Human Build 38 patch release 12; * Size of sum of minor contributing chromosomes.

	All	Group 1	Group 2	Group 3
Number of Scaffolds	2832	945	467	1420
Total scaffold size (Scaffold N50)	2904 Mb (44.85 Mb)	2,697 Mb (45.09 Mb)	165 Mb (13.74 Mb)	41 Mb (34.60 kb)
Size matched with GRCh38.p12 (%)	2851 Mb (98.2)	2691 Mb (99.8)	160 Mb (96.2)	0
by Sequence types Chromosomes (or alternative)	2839 Mb	2681 Mb	158 Mb	0
Fix	8047 kb	7831 kb	216 kb	0
Random	2783 kb	1906 kb	878 kb	0
Unknown chromosomes	1005 kb	648 kb	358 kb	0
Scaffolds matched multiple chromosomes of GRCh38.p12	487	343	144	0
Total size of scaffolds contributed from multiple chromosomes *	22.2 Mb	21.1 Mb	1.1 Mb	0

**Table 2:** The read counts of unmapped reads by samples.

Sample ID	Ancestry	Population	Total Number of Unmapped Reads (K)		Unpaired Reads, Counts (K) (%)	Mapped on AK1, Read Counts (K) * Mapping Rate (%)				Suggestive Microbial Origin, Read Count
						Overall		Mapping Quality > 10		
HG02922	AFR	Esan	59,751	Average 42,613	36,871 (61.7)	205 (0.9)	Mean % 0.90	110 (0.5)	Mean % 0.46	318
HG03052		Mende	34,958		21,174 (60.6)	127 (0.9)		67 (0.5)		401
NA19625		African-American SW	48,718		34,396 (70.6)	121 (0.8)		63 (0.4)		353
HG01879		African-Caribbean	35,674		198,064 (55.5)	165 (1.0)		78 (0.5)		1191
NA19017		Luhya	33,965		20,442 (60.2)	96 (0.7)		56 (0.4)		2188
HG00419	EAS	South. Han Chinese	34,935	Average 36,474	22,398 (64.1)	131 (1.0)	Mean % 0.95	66 (0.5)	Mean % 0.51	527
NA18525		Han Chinese	15,620		8,759 (56.1)	51 (0.7)		34 (0.5)		517
HG01595		Kinh Vietnamese	59,355		31,507 (53.1)	265 (1.0)		140 (0.5)		3405
NA18939		Japanese	27,950		15,520 (55.5)	127 (1.0)		66 (0.5)		522
HG00759		Dai Chinese	44,510		21,418 (48.1)	234 (1.0)		117 (0.5)		512
NA20502	EUR	Tuscan	26,343	Average 26,711	19,640 (74.6)	57 (0.9)	Mean % 0.88	33 (0.5)	Mean % 0.49	1557
HG00096		British	29,915		16,773 (56.1)	108 (0.8)		64 (0.5)		1878
HG01500		Spanish	31,331		15,726 (50.2)	164 (1.1)		76 (0.5)		2423
HG00268		Finnish	19,255		12,139 (63.0)	58 (0.8)		36 (0.5)		289
Total Average (Mean ± sd)			35,877 ± 13,193		21,184 ± 8091 (59.0%)	137± 65 (0.92%)		71 ±31 (0.49%)		1149 ± 988

Suggestive microbial origin was analyzed by GATK-pathSeq. African-American SW, African-American Southwes; $*\;\mathrm{Mapping}\;\mathrm{rate}=\frac{No.\;of\;reads\;re-aligned\;to\;AK1}{\left(total\;unmapped\;reads\;-\;unpaired\;read\right)}$.

**Table 3:** The distribution of repetitive sequences on the putative missing regions on AK1 scaffolds. The estimated missing regions by unmapped reads, 110 regions (≥X10, ≥2 indiv) and 38 regions (≥X10, ≥7 indiv), were investigated on the distribution of repetitive sequences with Repeat Masker. Mean% (SD).

Family		110 Regions (More than Ten Reads Are Mapped in More than Two Samples	38 Regions (More than Ten Reads Are Mapped in More than Seven Samples)
		Mean % (SD)	Mean % (SD)
SINE	All	8.01(9.85)	2.54 (5.41)
	ALUs	6.41 (12.25)	0.27 (1.63)
	MIRs	1.6 (6.65)	2.27 (7.37)
LINE	All	7.34 (13.35)	3.64 (13.80)
	LINE1	5.13 (15.50)	3.64 (13.80)
	LINE2	2.21 (10.77)	0
	L3/CR1	0	0
LTR	All	2.47(4.79)	0.56 (2.50)
	ERVL	0.88 (5.86)	0
	ERVL-MaLRs	0.98 (4.35)	0.56 (2.50)
	ERV-class I	0.60 (3.93)	0
	ERV-class II	0	0
DNA	All	0.14 (0.70)	0
	hAT-Charlie	0.14 (0.70)	0
	TcMar-Tigger	0	0
Unclassified		0.48 (5.01)	0
Small RNA		0.05 (0.51)	0
Satellite		8.94 (26.92)	7.85 (26.82)
Simple repeats		17.62 (33.73)	10.82 (29.95)
Low complexity		11.80 (31.59)	0.52 (2.00)

SINE = Short interspersed nuclear elements; MIR = Mammalian-wide interspersed repeats; LINE = Long interspersed nuclear elements; LTR = Long terminal repeat; ERVL = Endogenous retrovirus-L; ERVL-MaLRs = Endogenous retrovirus-L-Mammalian apparent LTR Retrotransposons; ERV = Endogenous retroviruses.

**Table 4:** Characteristics and verifications of the presence of the estimated globally missing regions on Group 1 scaffolds. The common candidate regions globally missing with ±2 kb of flanking sequences were searched and 20 of 31 globally missing regions (shared by ≥7 individuals) were verified by PCR.

AK1 Genome Information				Sequence Comparison Using UCSC BLAT			Validated by PCR				Hg38 Position	Verified Actual Indel Size (bp)	Breakpoint Structure	Mechanism	Microhomology (bp)	Microhomology Sequence or Homologous Sequence
ID	Scaffold of AK1	The Estimated Location of Globally Missing Region (≥7 indiv)		Human (GRCh38)	Chimp (panTro6)	Gorilla (gorGor4)	Eur 1	Eur 2	Eur 3	Eur 4						
		Start	End													
G1-1	KV784719.1	30,209,977	30,210,924	X	O	O	O	O	O	O	chr13:48910547-48914294	1198	Unique-Unique	NHEJ	0	
G1-2	KV784719.1	79,001,655	79,002,640	X	N	O	X	X	X	X	chr13:97337324-97340428	1333	SR-SR	NHEJ	4	TGTG
G1-3	KV784719.1	93,452,303	93,455,222	-	O	O	X	X	X	X	N/A	N/A	N/A	N/A	N/A	N/A
G1-4	KV784719.1	93,470,705	93,471,918	-	O	O	X	X	X	X	N/A	N/A	N/A	N/A	N/A	N/A
G1-5	KV784720.1	27,885,647	27,886,104	X	O	O	O	O	O	O	chr4:79781761-79785451	767	Alu-Unique	NHEJ	2	CT
G1-6	KV784723.1	8,349,171	8,349,628	X	O	O	O	O	O	O	chr4:181366776-181370046	1192	Unique-Unique	NHEJ	0	
G1-7	KV784723.1	10,288,012	10,288,493	X	O	O	Del	Del	Pol	Del	chr4:179430209-179433860	827	Unique-Unique	NHEJ	4	ATTT
G1-8	KV784723.1	34,400,763	34,401,227	X	O	O	X	X	X	X	chr4:155347518-155351075	901	Unique-Unique	NAHR	38	TTTCTTGTCTCCTGCCTTCTGCCAAGCCTTAGTCACAA
G1-9	KV784731.1	15,610,509	15,611,959	X	O	N	O	O	O	O	chr5:6446724-6450554	1636	SR-Unique	NHEJ	4	CTGC
G1-10	KV784736.1	6,179,476	6,184,176	X	O	O	O	O	O	O	chr6:67607329-67611067	4961	Alu-L1	NHEJ	4	AAAA
G1-11	KV784736.1	18,433,040	18,435,697	X	O	O	O	O	O	O	chr6:79899617-79903449	2892	Unique-Unique	NHEJ	5	GGACT
G1-12	KV784738.1	33,432,222	33,432,240	X	O	N	X	X	X	X	chr10:2389608-2395439	4163	Unique-Unique	NHEJ	5	CCCTC
G1-13	KV784747.1	1,225,842	1,227,344	X	O	O	Del	Del	Pol	O	chr6:28174388-28177850	2035	Unique-Unique	NHEJ	2	AG
G1-14	KV784754.1	50,234,036	50,235,663	X	O	O	O	O	O	O	chr8:136025060-136028726	1957	Unique-Alu	NHEJ	5	ATCTC
G1-15	KV784761.1	2,374,855	2,374,857	X	-	-	O	O	O	O	chr18:13980325-13983782	543	Unique-Unique	NHEJ	4	TCCT
G1-16	KV784762.1	646,396	646,455	X	N	N	X	X	X	X	chr19:869056-876703	2372	G-rich-G-rich	NHEJ	4	GGGG
G1-17	KV784762.1	942,159	943,260	X	O	O	O	O	O	O	chr19:1160489-1162472	3127	Alu-Alu	NAHR	25	CCTGTAATCCCAGCACTTTGGGAGG
G1-18	KV784774.1	387,226	387,651	X	O	O	X	X	X	X	chrX:47084676-47092500	2920	SR-Alu	NHEJ	3	ATG
G1-19	KV784797.1	27,753,978	27,754,392	X	O	O	O	O	O	O	chr1:93874952-93876859	2521	Unique-Alu	NHEJ	0	
G1-20	KV784800.1	13,617,523	13,617,941	X	O	O	Pol	Pol	O	Pol	chr10:63781277-63784929	763	Alu-Unique	NHEJ	4	AGAA
G1-21	KV784803.1	15,594,978	15,595,455	X	O	O	O	Pol	Del	Del	chr14:88710100-88713185	1390	LTR-LTR	NHEJ	6	GAACTG
G1-22	KV784803.1	21,188,206	21,188,829	X	O	O	Del	Del	O	O	chr14:83119034-83122153	1504	L1-Unique	NHEJ	3	AGA
G1-23	KV784804.1	4,078,861	4,078,900	X	O	O	O	O	O	O	chr17:40521389-40524617	820	Unique-Alu	NHEJ	1	G
G1-24	KV784806.1	65,330,325	65,332,270	X	O	O	O	O	O	O	chr2:21821760-21825542	2160	L1-Unique	NHEJ	1	T
G1-25	KV784811.1	3,734,091	3,735,143	X	O	O	O	O	O	O	chr7:68760760-68763395	2414	Alu-Unique	NHEJ	3	AAG
G1-26	LPVO02000186.1	2,132,760	2,132,810	X	O	O	Pol	Pol	O	Pol	chr3:95822539-95830080	2497	L1-Unique	NHEJ	0	
G1-27	LPVO02000191.1	8,716,140	8,716,258	X	O	N	X	X	X	X	chr3:194273873-194277269	720	G-rich-G-rich	NHEJ	2	GG
G1-28	LPVO02000230.1	3,020,537	3,020,573	X	X	N	X	X	X	X	chr5:181099166-181102877	615	SR-SR	NHEJ	3	CCT
G1-29	LPVO02000423.1	11,658,530	11,658,908	X	O	O	X	X	X	X	chr11:101923894-101927461	806	Alu-Alu	NHEJ	8	GTGCAGTG
G1-30	LPVO02000423.1	13,811,264	13,811,292	X	O	O	Del	Pol	Del	Pol	chr11:104076897-104080443	579	LTR-Unique	NHEJ	2	TT
G1-31	LPVO02000621.1	1,217,413	1,217,481	X	N	N	X	X	X	X	chrX:2318537-2323680	4923	Alu-Alu	NAHR	24	GTGGAGGTTGCAGTGAGCCGAGAT

The Estimated Location of Globally Missing Region start/end (≥7 indiv) = Start/End postion of the sequence mapped by unmapped reads of more 7 samples; Eur, European;X, Not exist; O, Same as AK1; “-“, Not matched to the primate reference genome; N, Matched but ambiguous sequences (Ns) were included; Del, Deletion; Pol, Polymorphic; SR, Simple Repeat; NHEJ, Non-homologous end-joining; NAHR, Non-allelic homologous recombination; N/A = Not available.

## Supplementary Materials

The following are available online at https://www.mdpi.com/2073-4425/11/11/1350/s1, Figure S1. The distribution of the read count proportions by read quality of the mapped reads and unmapped reads on each of the two genome assemblies; Figure S2. The distribution of the read counts by read quality; Figure S3. The 31 globally missed regions (shared by ≥7 individuals) by visual inspection with UCSC genome browser and IGV; Table S1. The match % between scaffolds and GRCh38 applied with different parameter sets; Table S2. The distribution of non-repetitive and repetitive sequences between GRCh38 genoms and AK1 Group 3 scaffolds by Repeat Masker.; Table S3. A total of 1390 regions on non-overlapping AK1 genome (>200 bp) of Group 1 and 2 were predicted to be putative coding regions; Table S4. Average mapping quality and average depth of mapped reads on GRCh38 and AK1; Table S5. The distribution of repetitive sequences on reference genome (GRCh38) and sequencing reads from 14 samples by Repeat masker; Table S6. The breadth of coverage and read counts by groups of AK1; Table S7. The 110 regions not on GRCh38 reference of Group 1, 2, and 3, including the regions with more than ten reads of more than two samples and the 64 similar sequences of 110 on BLASTn search; Table S8. The putative proteins of translated BLAST search on the 25 of 110 regions on Group 1, 2, and 3, where more than ten reads are mapped in more than two samples; Table S9. The list and translated BLAST search of 38 globally missing regions where more than ten reads are mapped in more than seven samples.

## Data and Materials Availability

When we compared between GRCh 38.p12 and AK1 genome, the chain file was used and supported the findings of this study. The chain file between GRCh 38.p12 and AK1 genome can be found at http://dx.doi.org/10.5281/zenodo.3921981.
