# Supplementary material for: A High Quality Asian Genome Assembly Identifies Features of Common Missing Regions
Source: Genes (Basel). 2020 Nov 13;11(11):1350. doi: 10.3390/genes11111350 (PMC7697454; doi:10.3390/genes11111350)
Supplement: Supplementary file 1 [file genes-11-01350-s001.zip › Supplemental_Figures_HighResolution_genes_FS1-S3.pptx]

## Slide 1
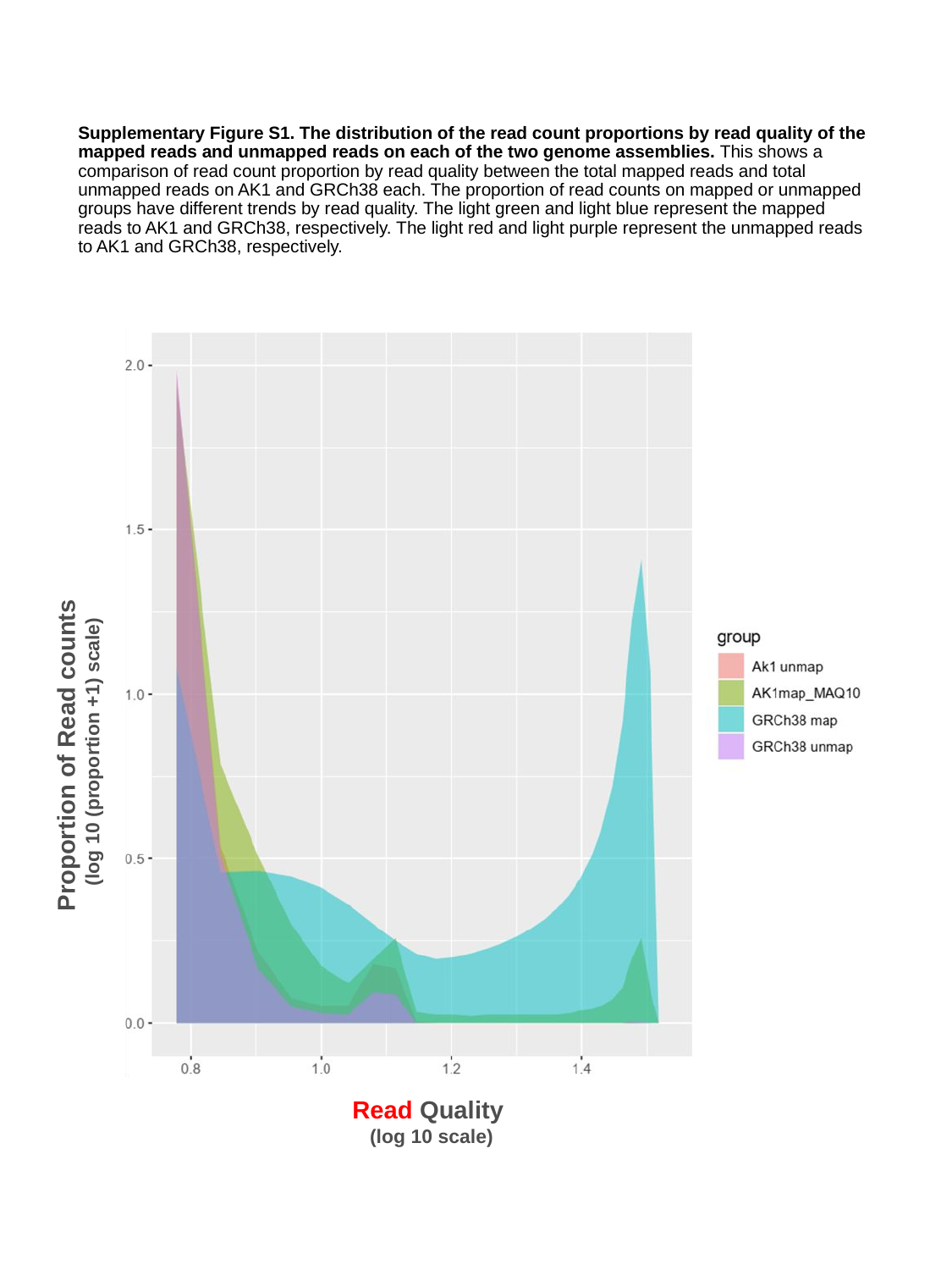

# Supplementary Figure S1. The distribution of the read count proportions by read quality of the mapped reads and unmapped reads on each of the two genome assemblies. This shows a comparison of read count proportion by read quality between the total mapped reads and total unmapped reads on AK1 and GRCh38 each. The proportion of read counts on mapped or unmapped groups have different trends by read quality. The light green and light blue represent the mapped reads to AK1 and GRCh38, respectively. The light red and light purple represent the unmapped reads to AK1 and GRCh38, respectively.
Proportion of Read counts
(log 10 (proportion +1) scale)
Read Quality
(log 10 scale)

## Slide 2
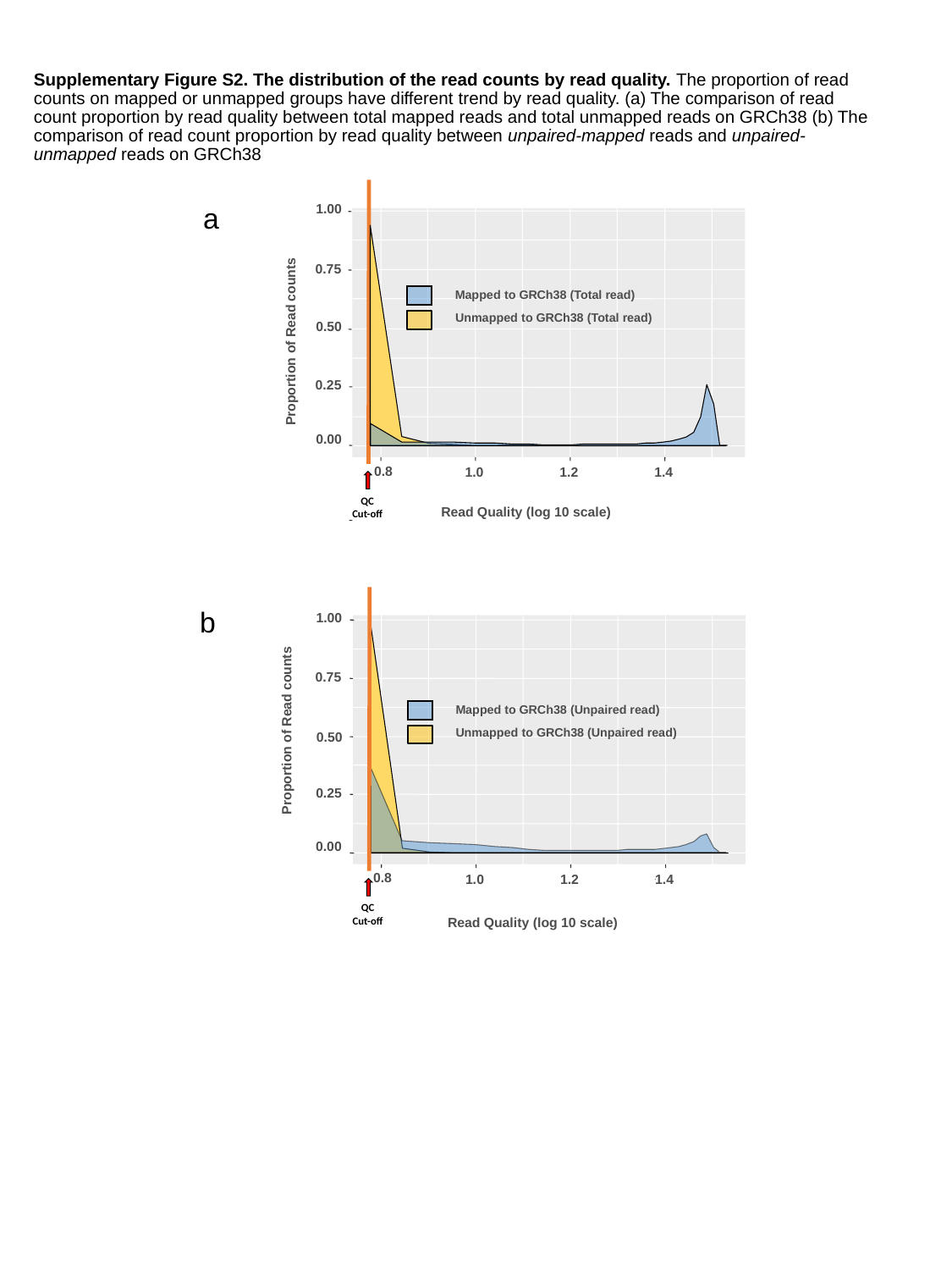

Supplementary Figure S2. The distribution of the read counts by read quality. The proportion of read counts on mapped or unmapped groups have different trend by read quality. (a) The comparison of read count proportion by read quality between total mapped reads and total unmapped reads on GRCh38 (b) The comparison of read count proportion by read quality between unpaired-mapped reads and unpaired-unmapped reads on GRCh38
1.00
0.75
Mapped to GRCh38 (Total read)
Unmapped to GRCh38 (Total read)
0.50
Proportion of Read counts
0.25
0.00
0.8
1.0
1.2
1.4
QC
Cut-off
Read Quality (log 10 scale)
a
1.00
0.75
.
Proportion of Read counts
0.50
0.25
0.00
0.8
1.0
1.2
1.4
QC
Cut-off
Read Quality (log 10 scale)
Mapped to GRCh38 (Unpaired read)
Unmapped to GRCh38 (Unpaired read)
b

## Slide 3
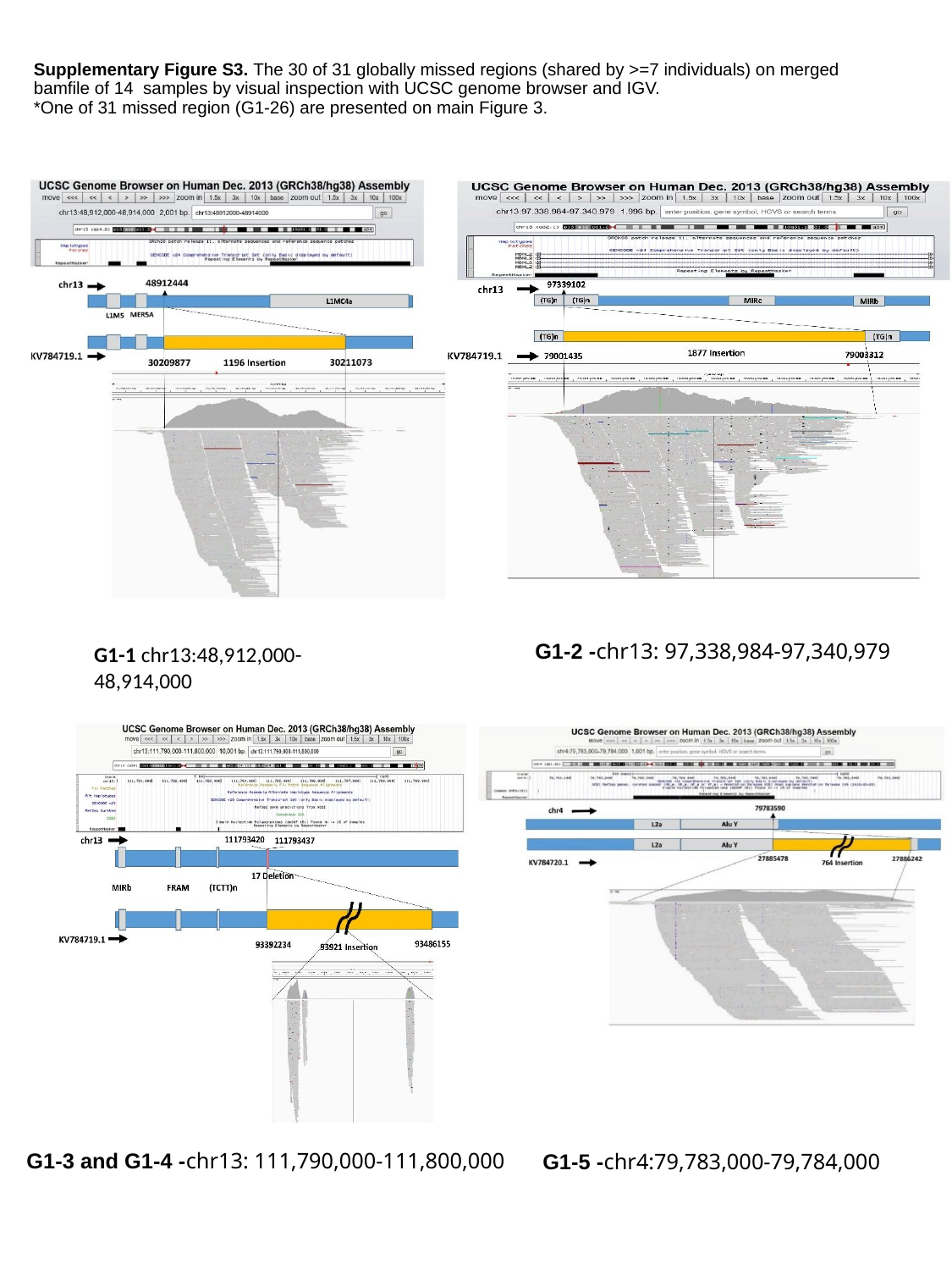

Supplementary Figure S3. The 30 of 31 globally missed regions (shared by >=7 individuals) on merged bamfile of 14 samples by visual inspection with UCSC genome browser and IGV.
*One of 31 missed region (G1-26) are presented on main Figure 3.
G1-2 -chr13: 97,338,984-97,340,979
G1-1 chr13:48,912,000-48,914,000
G1-3 and G1-4 -chr13: 111,790,000-111,800,000
G1-5 -chr4:79,783,000-79,784,000

## Slide 4
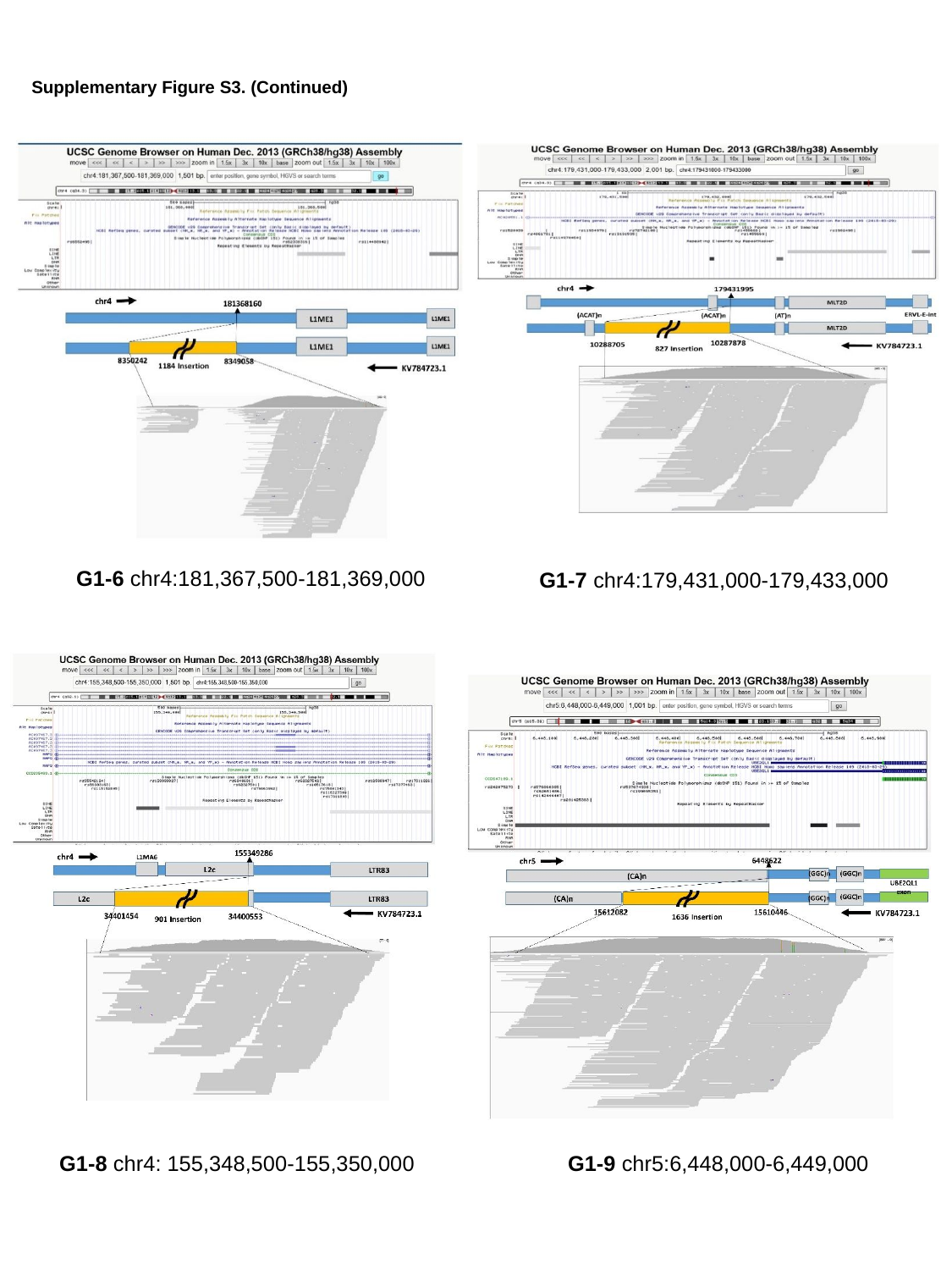

Supplementary Figure S3. (Continued)
G1-6 chr4:181,367,500-181,369,000
G1-7 chr4:179,431,000-179,433,000
G1-8 chr4: 155,348,500-155,350,000
G1-9 chr5:6,448,000-6,449,000

## Slide 5
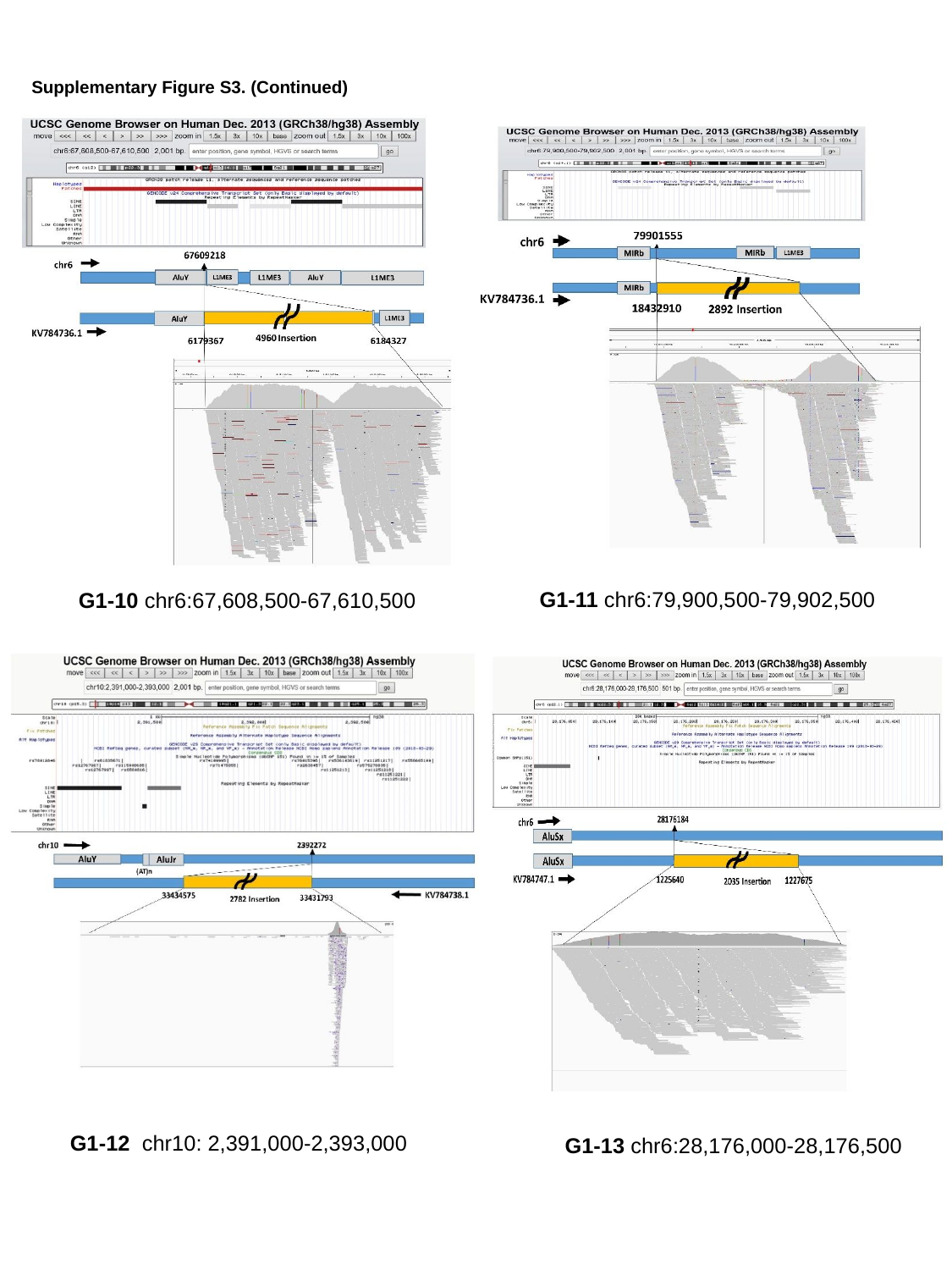

Supplementary Figure S3. (Continued)
G1-11 chr6:79,900,500-79,902,500
G1-10 chr6:67,608,500-67,610,500
G1-12 chr10: 2,391,000-2,393,000
G1-13 chr6:28,176,000-28,176,500

## Slide 6
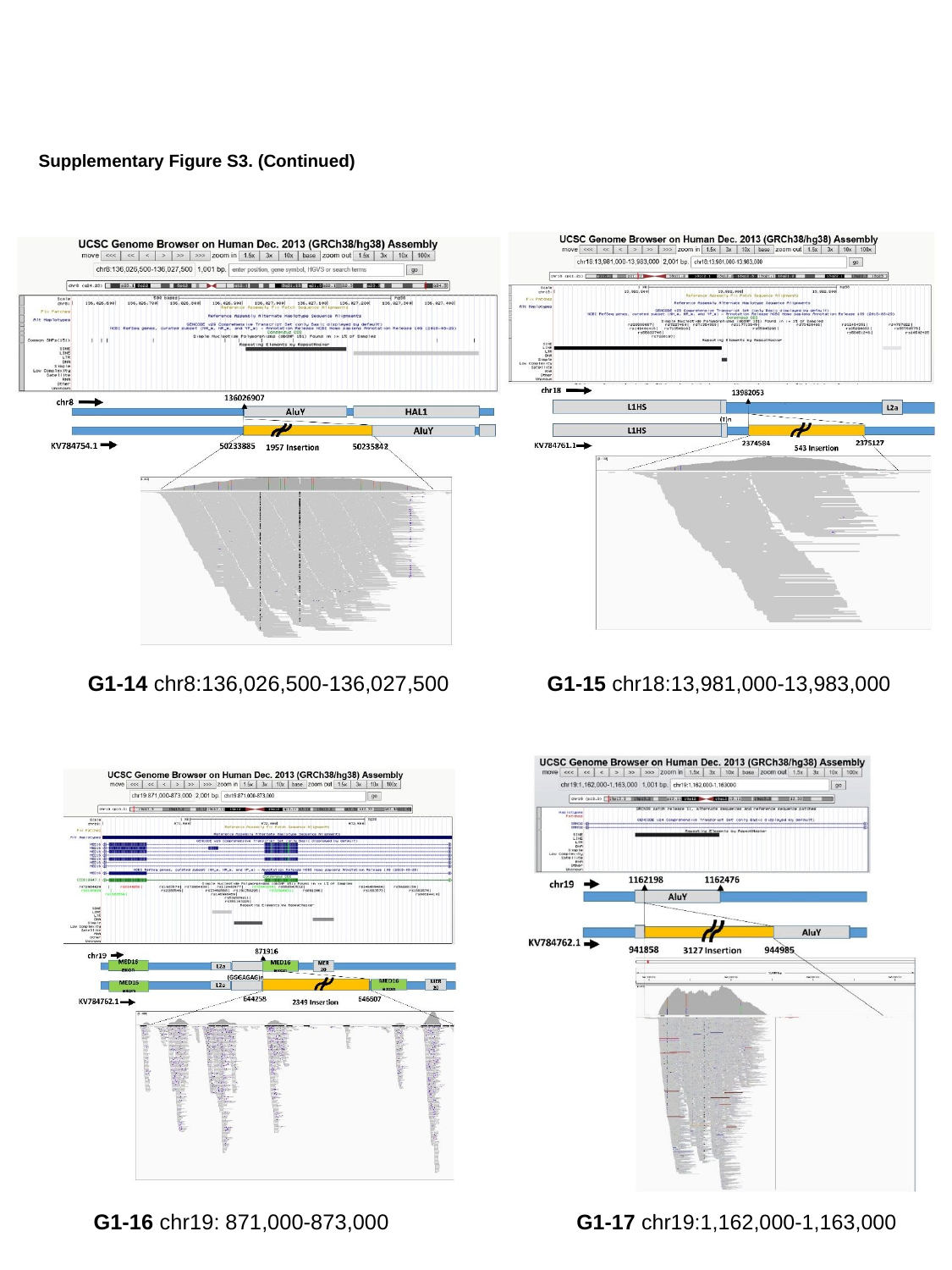

Supplementary Figure S3. (Continued)
G1-14 chr8:136,026,500-136,027,500
G1-15 chr18:13,981,000-13,983,000
G1-16 chr19: 871,000-873,000
G1-17 chr19:1,162,000-1,163,000

## Slide 7
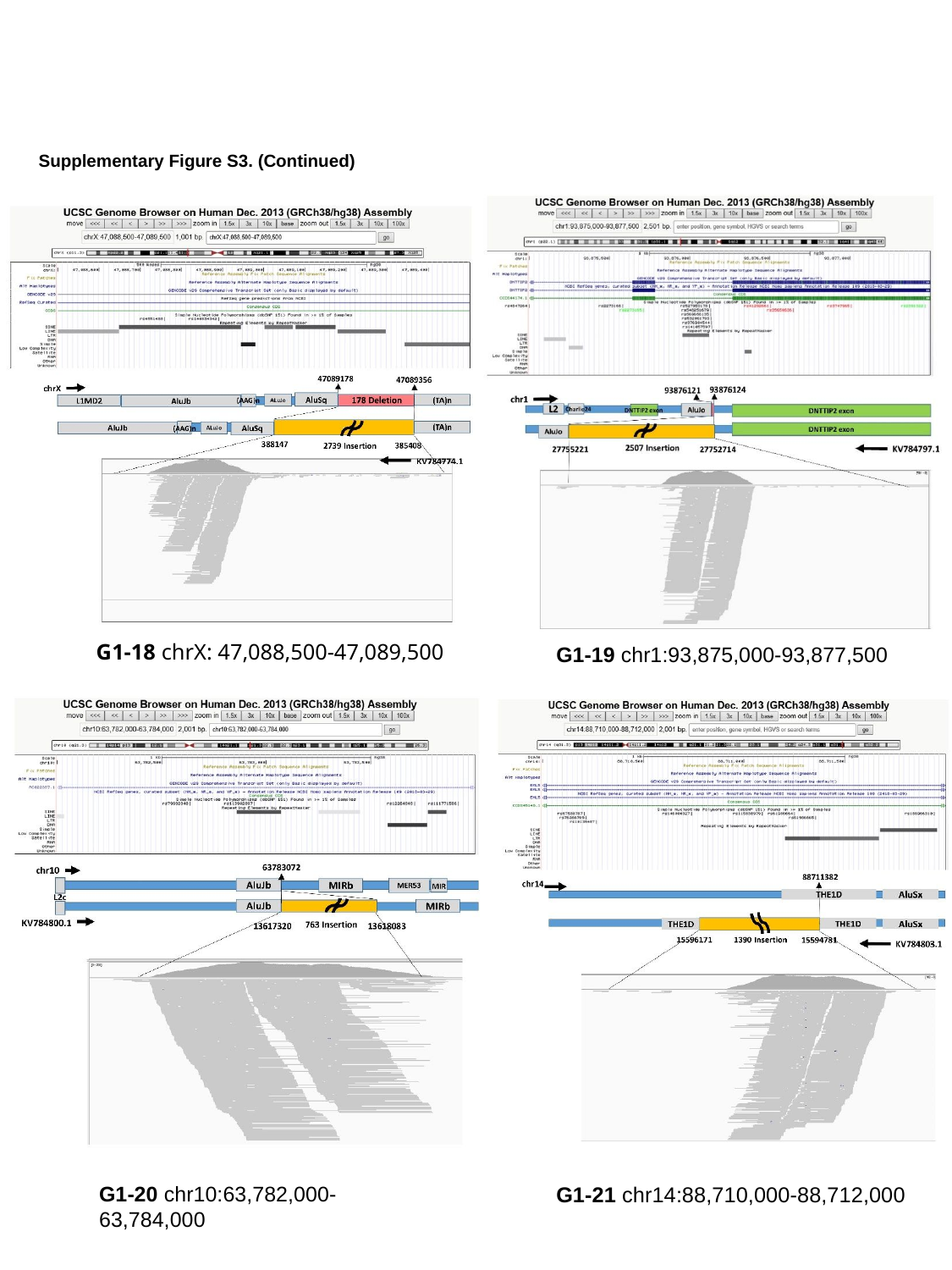

Supplementary Figure S3. (Continued)
G1-18 chrX: 47,088,500-47,089,500
G1-19 chr1:93,875,000-93,877,500
G1-20 chr10:63,782,000-63,784,000
G1-21 chr14:88,710,000-88,712,000

## Slide 8
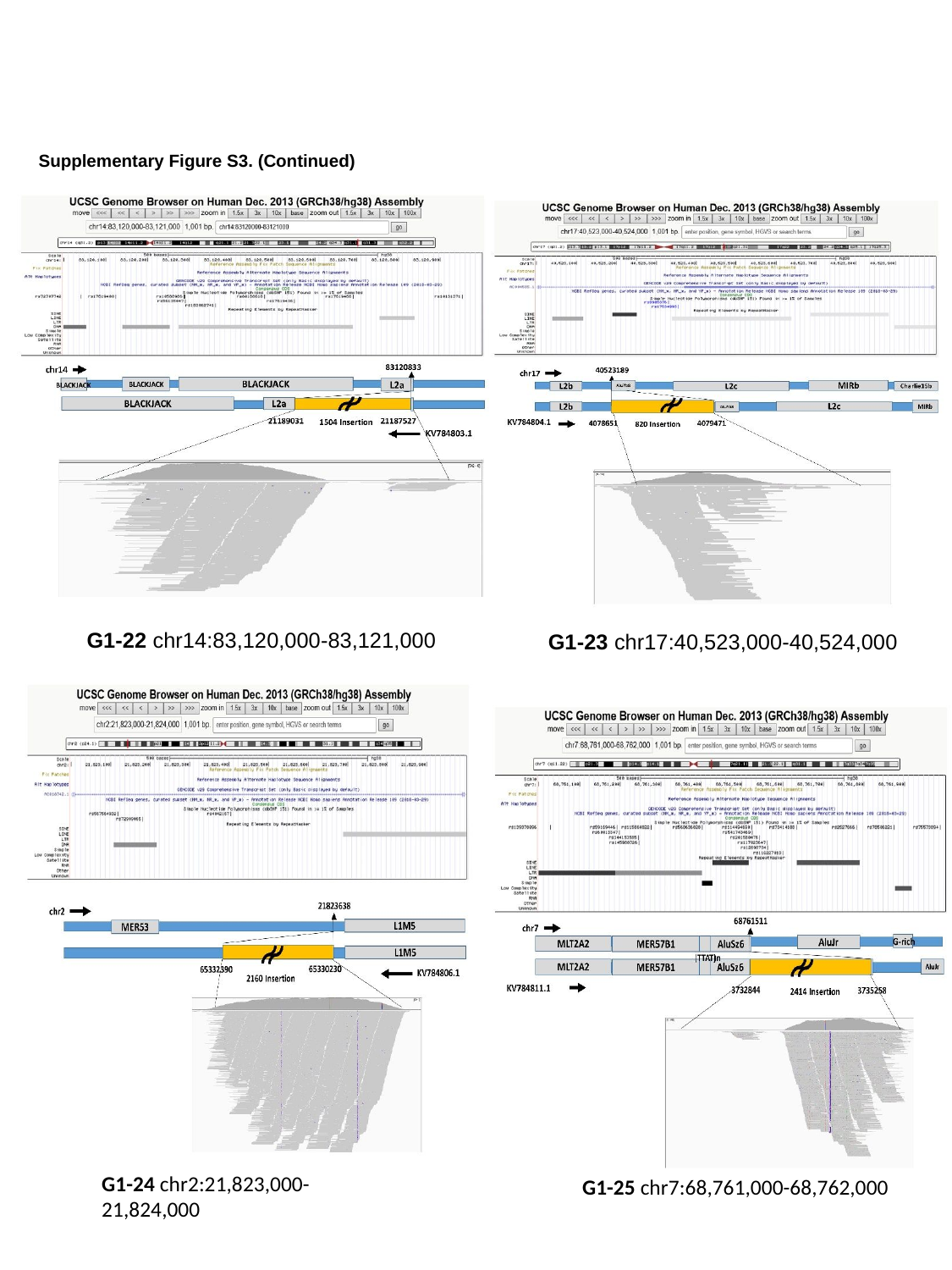

Supplementary Figure S3. (Continued)
G1-22 chr14:83,120,000-83,121,000
G1-23 chr17:40,523,000-40,524,000
G1-24 chr2:21,823,000-21,824,000
G1-25 chr7:68,761,000-68,762,000

## Slide 9
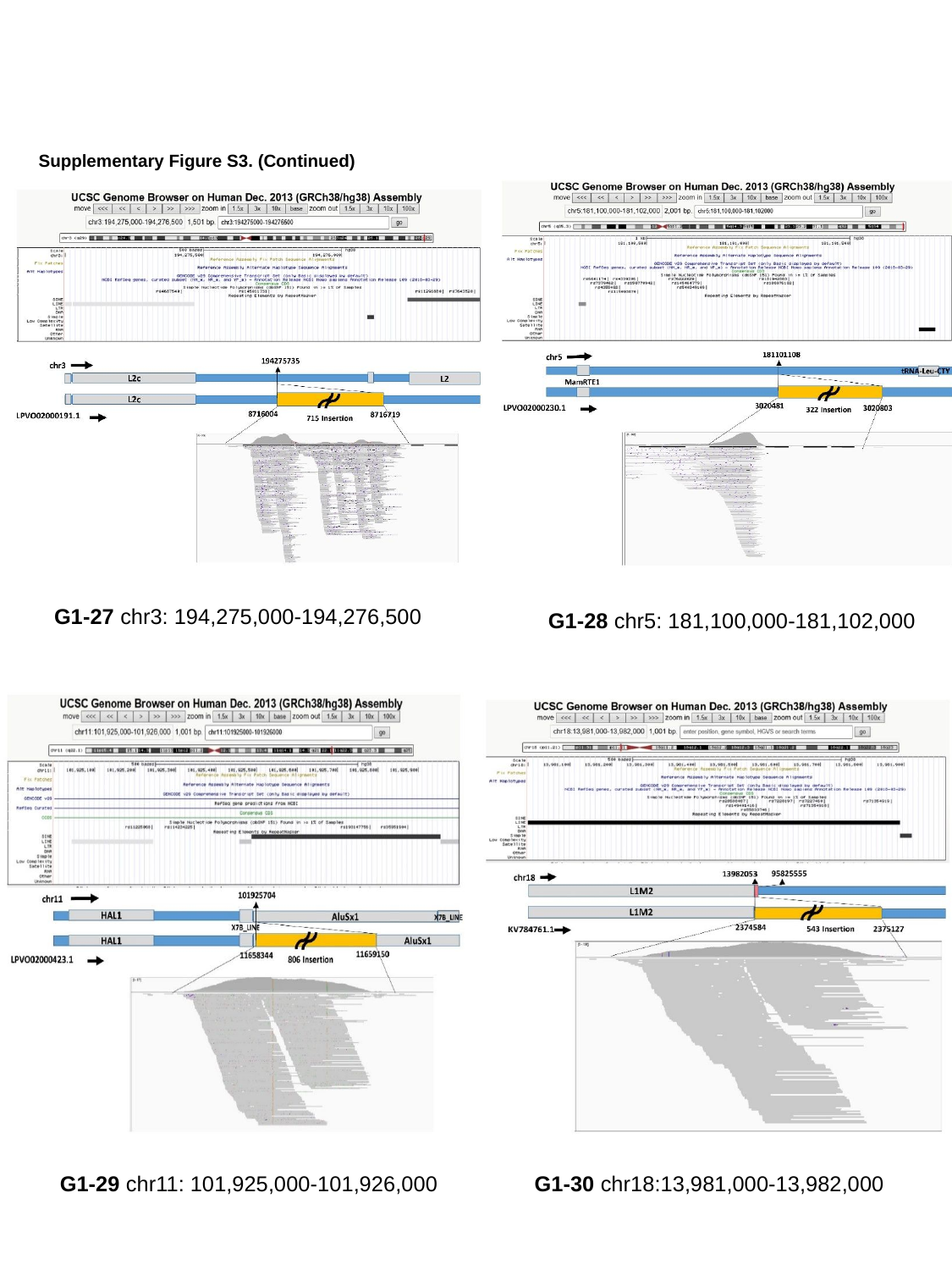

Supplementary Figure S3. (Continued)
G1-27 chr3: 194,275,000-194,276,500
G1-28 chr5: 181,100,000-181,102,000
G1-29 chr11: 101,925,000-101,926,000
G1-30 chr18:13,981,000-13,982,000

## Slide 10
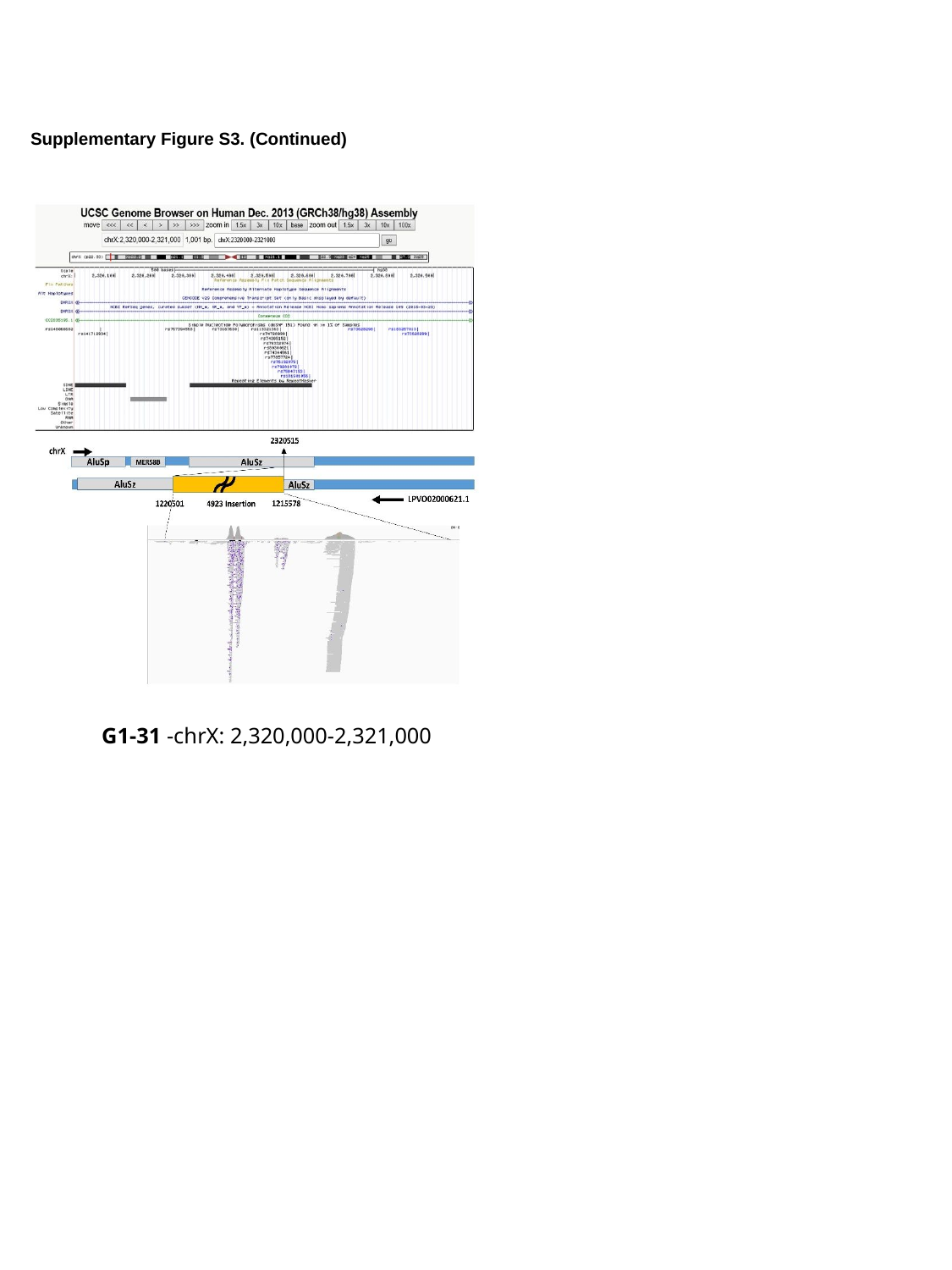

Supplementary Figure S3. (Continued)
G1-31 -chrX: 2,320,000-2,321,000
