# Supplementary figures and images for: A High Quality Asian Genome Assembly Identifies Features of Common Missing Regions
Source: Genes (Basel). 2020 Nov 13;11(11):1350. doi: 10.3390/genes11111350 (PMC7697454; doi:10.3390/genes11111350)

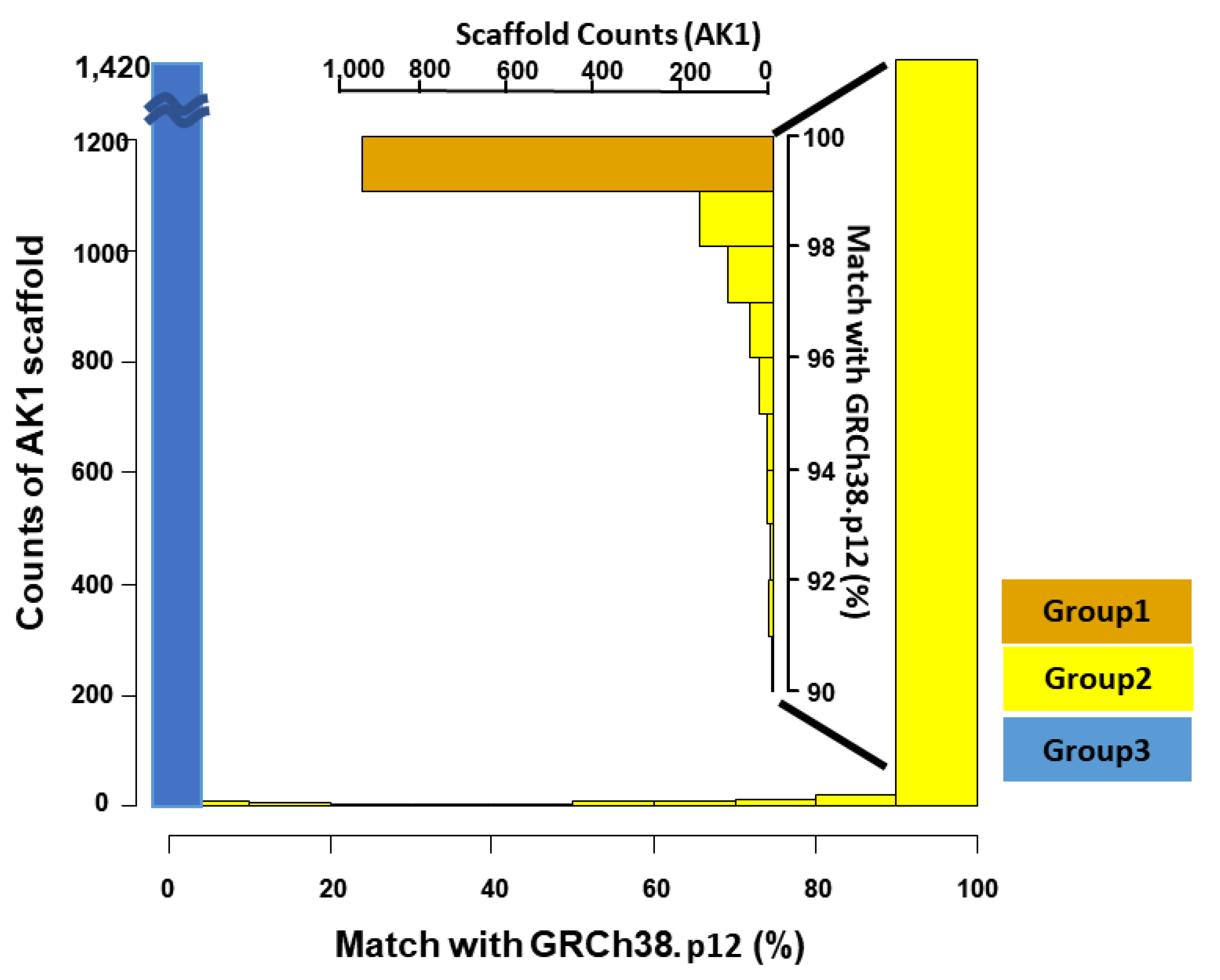

Supplement: Supplementary file 1 [file genes-11-01350-s001.zip › genes-976896-g001.tif]

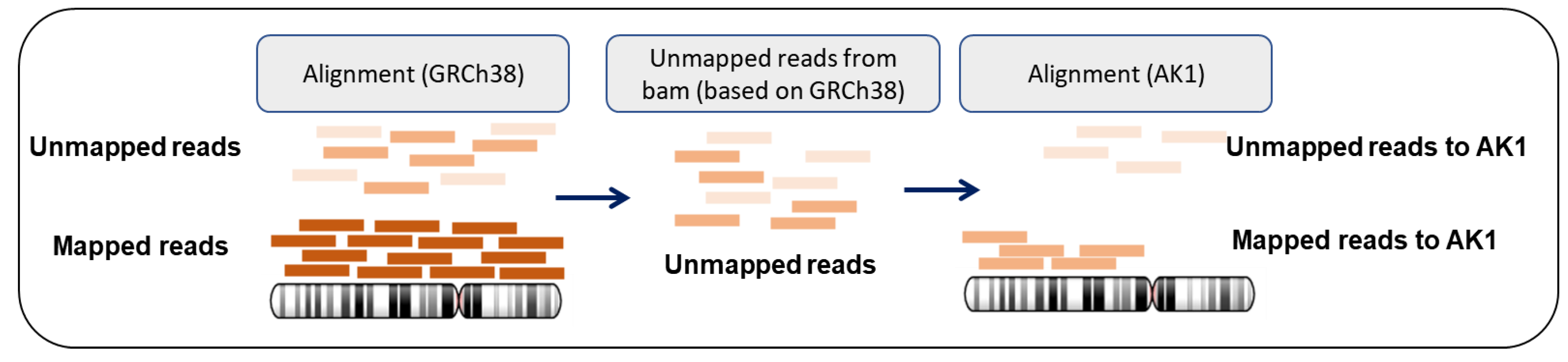

Supplement: Supplementary file 1 [file genes-11-01350-s001.zip › genes-976896-g002.tif]

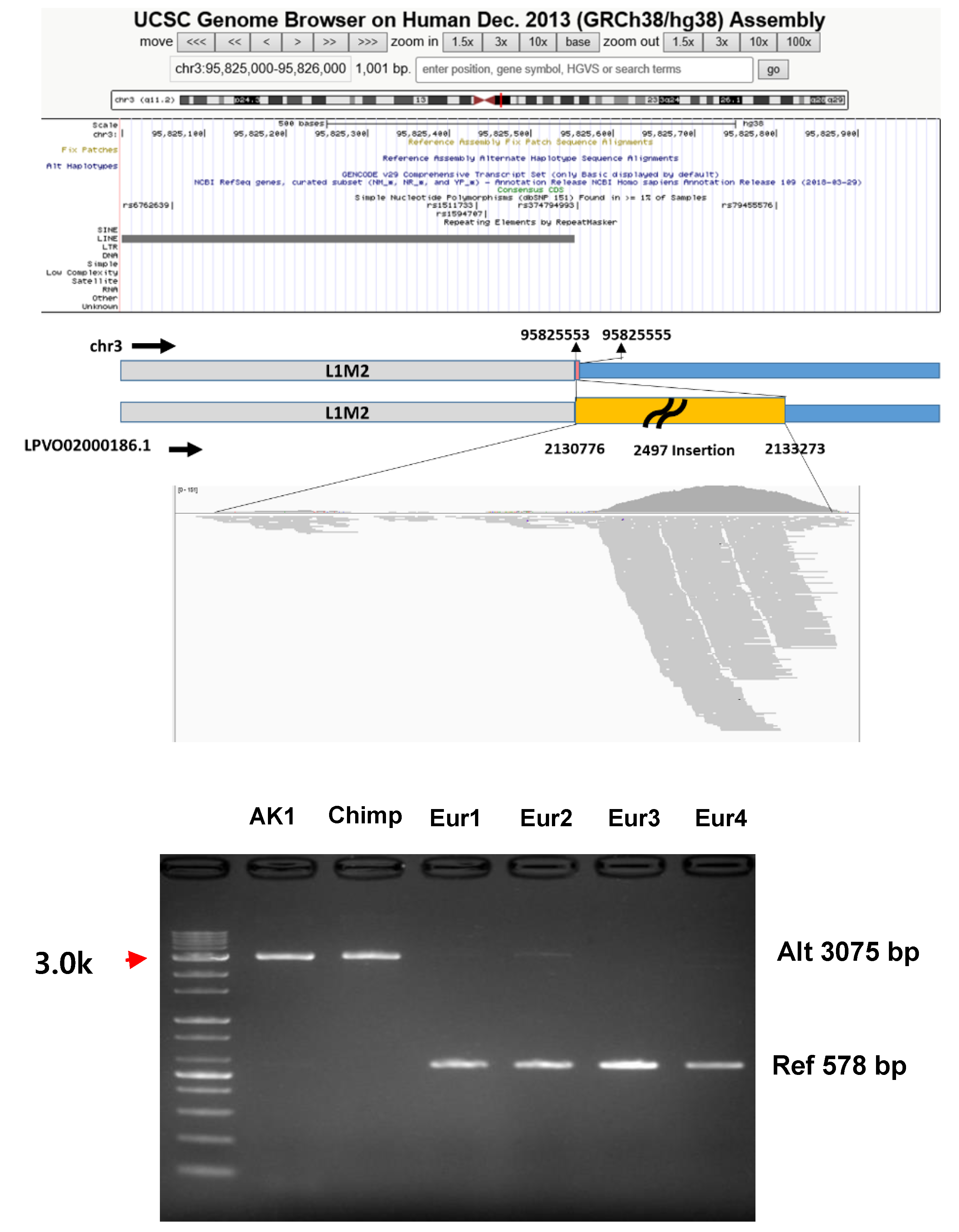

Supplement: Supplementary file 1 [file genes-11-01350-s001.zip › genes-976896-g003.tif]
